# Duration of Systole and Diastole for Hydrodynamic Testing of Prosthetic Heart Valves: Comparison Between ISO 5840 Standards and *in vivo* Studies

**DOI:** 10.5935/1678-9741.20160036

**Published:** 2016

**Authors:** Ovandir Bazan, Jayme Pinto Ortiz

**Affiliations:** 1 Department of Mechanical Engineering, Escola Politécnica of the University of São Paulo (EPUSP), Brazil

**Keywords:** Ventricular Function, Left, Heart valve prosthesis, *In Vitro* Techniques, Heart Rate

## Abstract

**Objective:**

To complement the ISO 5840 standards concerning the duration of left ventricular systole and diastole as a function of changes in heart rates according to in vivo studies from the physiologic literature review.

**Methods:**

The systolic and diastolic durations from three *in vivo* studies were compared with the durations of systole proposed by the ISO 5840:2010 and ISO 5840-2:2015 for hydrodynamic performance assessment of prosthetic heart valves.

**Results:**

Based on the *in vivo* studies analyzed, the systolic durations proposed by the ISO 5840 standard seemed consistent for 45 and 120 beats per minute (bpm), and showed diverse results for the 70 bpm condition.

**Conclusion:**

Information on the realistic validation of the operation of left ventricular models for different heart rates were obtained.

**Table:** 

Abbreviations, acronyms & symbols	
Bpm	= Beats per minute
CO	= Cardiac output
ECG	= Electrocardiogram
FDA	= U. S. Food and Drug Administration
HR	= Heart rate
PCG	= Phonocardiogram
QS_2_	= Total electromechanical systole
QT	= Electrical systole
RR	= Total cardiac cycle duration

## INTRODUCTION

Cardiac simulators or pulse duplicator systems are required to the hydrodynamic performance analysis of prosthetic heart valves (*i.e.*, mechanical, biological, and synthetic prostheses), in which pulsatile flow testing was established by the ISO 5840 standard and the U.S. Food and Drug Administration (FDA) draft guidance^[1-4]^. Therefore, the main purpose of these *in vitro* experiments is the realistic simulation of the left human heart, under conditions of similarity. Besides geometry and flexibility (anatomical conditions), left ventricular models are expected to operate according to physiological (or pathological) characteristics, for instance, arterial impedance, dynamic viscosity of the blood analog fluid, intraventricular and systemic pressures, end-diastolic volume, stroke volume, cardiac output (CO), heart rates (HRs) and related duration of left ventricular systole and diastole. Since these variables are changed, different mitral and aortic pressures and flow conditions are imposed through the prostheses, in which their own type, position, dimension, and dynamic behavior are implicated.

The ISO 5840 offers some guidelines for the test apparatus requirements and specifies the procedures concerning the hydrodynamic performance testing. As minimum performance requirements for the pulsatile-flow regime, the condition of 70 cycles/min with systolic duration of 35% is required^[3,5]^. However, the duration of systole and diastole reduces as HR increases, but nonlinearly. Based on *in vivo* data, as HR increases, the reduction is much more pronounced in the diastole phase^[6,7]^.

The purpose of this work was to complement the ISO 5840 guidance for the experimental validation of pulse duplicator systems besides the rest condition, based on *in vivo* data concerning the duration of systole and diastole as a function of changes in HRs.

## METHODS

The duration of left ventricular systole and diastole concerning normal healthy people in response to increasing HR were examined according to *in vivo* data^[6-9]^ in order to allow a comparison with the data suggested by the ISO 5840:2010 and ISO 5840-2:2015 standards^[3,5]^.

It is important to note that, since the purpose of this study was to provide data that can be applied to the dynamic operation of left ventricular models, the physiological literature review was focused on electromechanical duration. Therefore, the (total) electromechanical systole (QS_2_) was assumed, instead of the electrical systole (QT)^[9]^. QS_2_ is the interval obtained from the Q wave on the electrocardiogram (ECG) to the second heart sound (S2) on the simultaneous phonocardiogram (PCG)^[8]^, or the duration of both isovolumetric contraction and left ventricular ejection phases. The mechanical duration of diastole is defined as total cardiac cycle duration (RR) minus the QS_2_.

The data from Husmann et al.^[6]^, Chung et al.^[8]^, and Boudoulas et al.^[9]^, respectively, are based on 30 subjects (mean age, 59.9 years), 25 subjects (mean age, 24 years), and 20 males (mean age, 40 years).

**Fig. 1:**
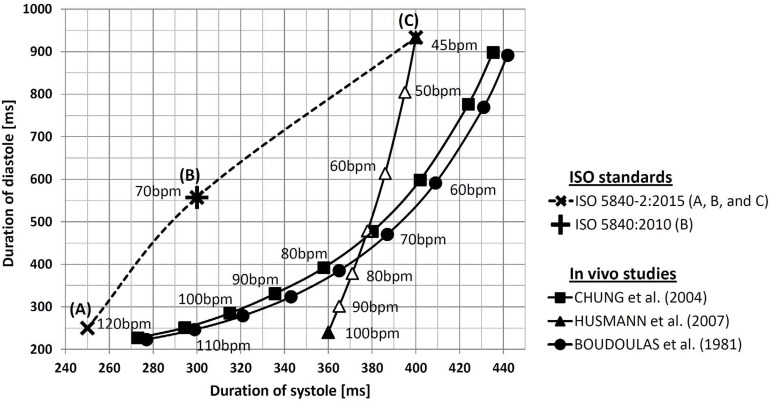
Duration of systole and diastole as a function of changes in HR according to some authors^[6,8,9]^, and the ISO 5840 standard. The data from Husmann et al.^[6]^ were explicit only for 45 and 100 bpm (black spots).

Figure 1 shows the duration of systole and diastole as a function of changes in HR according to some authors^[6-9]^ and the recommendation of the ISO 5840 standard^[3,5]^.

The testing conditions suggested by the ISO 5840-2:2015 and ISO 5840:2010 were plotted. Systolic duration of 35% for 70 beats per minute (bpm) is the same for both^[3,5]^. Since the data from Husmann et al.^[6]^ were explicit only for 45 and 100 bpm (with black spots), white spots were placed according a previous assessment in which the duration of the diastole declines asymptotically as HR increases^[7]^.

## DISCUSSION

Concerning the pulsatile-flow analysis, the ISO 5840 establishes the testing at 70 bpm with systolic duration of 35% as minimum performance requirements^[3,5]^ (Figure 1, dashed line). This criteria has been adopted in several *in vitro* studies^[10-12]^, yet not always: Akagawa et al.^[13]^, for instance, performed their study at 80 bpm with a systolic duration of 40%.

The hydrodynamic performance assessment of prosthetic heart valves including different HRs allows important information concerning the dynamic behavior of the prostheses. The ISO 5840 suggests, for instance, three simulated low, normal, and high HRs for the regurgitant volumes (reverse flow) measurement^[3,5]^. In this sense, besides the 70 bpm condition, left ventricular systolic duration was proposed by the ISO 5840 also for 45 and 120 bpm (respectively systolic duration of 30% and 50%) in its last release (Figure 1, dashed line, ISO 5840-2:2015), September 2015. Concerning valve substitutes implanted by transcatheter techniques, the ISO 5840-3:2013 established different systolic durations (from 50 to 35%) only as a function of 5 pediatric subpopulations (from birth to 22 years), including three simulated low, normal, and high HRs for each systolic ratio^[4]^.

The data concerning systolic or diastolic duration from three *in vivo* studies were included in Figure 1. Husmann et al.^[6]^ found that the length of systole shortened from 400 to 360 ms, respectively, for 45 to 100 bpm. This result shows that the duration of systole lengthened from 30% to 60% of the cardiac cycle^[6]^. The data from Chung et al.^[8]^ and Boudoulas et al.^[9]^ were quite similar, specially as HR increases. The percentage duration of systole lengthened from 35% to 54% and from 36% to 54% of the cardiac cycle, respectively, in the study by Chung et al.^[8]^ and Boudoulas et al.^[9]^, cases with mean age of 24 and 40 years.

According to Figure 1, the *in vivo* studies^[6,8,9]^ showed diverse results if compared with the suggestion of the ISO 5840 standard for the 70 bpm condition, systolic duration of 35%^[3,5]^. However, specifically at 45 and 120 bpm, respectively the systolic duration of 30% and 50% (Figure 1, C and A) seemed consistent with the *in vivo* results.

Regarding the hydrodynamic characterization of prosthetic heart valves including different HRs, we consider that realistic *in vitro* simulations should be similar with the range of systolic (and diastolic) duration according to *in vivo* studies^[6,8,9]^. Furthermore, taking into account that the wave responses from left ventricular models include their dynamics, they should be validated for the electromechanical systole and diastole intervals^[9]^, as exposed in the methodology. However, in terms of general hydrodynamic performance evaluation, valid analyses are possible for diverse experimental setups, left ventricular models, and also specific simulated ventricular states. However, each of these variables influences the flow, and the behavior of a prosthetic valve depends on its specific simulated conditions.

The ISO 5840:2010 also addresses the operational environment of the prosthetic heart valves for pathological conditions, in terms of arterial systolic and diastolic pressures^[2,4,5]^. Patients with heart disease do not generally produce normal pressure and flow responses. In this sense, left ventricular models should be validated properly, considering the in vivo pathological waveforms. For instance, patients with heart failure are characterized by an extended left ventricular systole and, consequently, a shorter interval of left ventricular diastole, in which the cardiac filling could be compromised^[14]^.

## CONCLUSION

The *in vivo* results from the literature review concerning systolic and diastolic duration based on the electromechanical intervals represent important information in order to realistically validate the operation of left ventricular models for different HRs.

Based on the *in vivo* studies analyzed, the systolic durations proposed by the ISO 5840 standard seemed consistent for 45 and 120 bpm, and showed diverse results for the 70 bpm condition.

**Table:** 

Authors' roles & responsibilities	
OB	Conception and design study; analysis and/or data interpretation; manuscript writing or critical review of its content; final manuscript approval
JPO	Final manuscript approval
